# Five-Year Risk of CIN3+ After CIN1 Biopsy in a Norwegian Screening Setting: Comparison of CIN1 Diagnosed in a Single Calendar Year and in Two Consecutive Calendar Years

**DOI:** 10.3390/pathogens15060657

**Published:** 2026-06-22

**Authors:** Sveinung Wergeland Sørbye, Mona Antonsen, Elin Richardsen

**Affiliations:** Department of Clinical Pathology, University Hospital of North Norway, 9006 Tromso, Norway; mona.antonsen@unn.no (M.A.); elin.richardsen@unn.no (E.R.)

**Keywords:** CIN1, cervical biopsy, CIN2+, CIN3+, cervical precancer, risk stratification, long-term follow-up, conservative management, cervical cancer screening, registry-based cohort

## Abstract

Cervical intraepithelial neoplasia grade 1 (CIN1) is usually managed conservatively, but uncertainty remains about the subsequent risk of clinically significant high-grade disease, particularly after repeated CIN1. We conducted a retrospective population-based cohort study using anonymized cervical cytology, HPV, and histopathology records from Northern Norway from 2011 to 2025. We described temporal trends in screening-related outcomes and estimated the 5-year risk of CIN3+ after histologically confirmed CIN1 diagnosed in a single calendar year or in two consecutive calendar years. Across 2011–2025, the annual datasets comprised 334,471 screening records; 35,796 had ASC-US+ cytology (10.7%), 29,723 had a positive HPV test (8.9%), 35,416 underwent biopsy (10.6%), and 7870 were diagnosed with CIN2+ (2.4%). HPV positivity increased from 0.9% in 2011 to 15.7% in 2025, whereas CIN2+ detection peaked at 3.1% in 2018 and declined to 1.8% in 2025. In person-based analyses, the 5-year risks after CIN1 diagnosed in a single calendar year versus two consecutive calendar years were 4.3% versus 3.4% for CIN3+, 0.2% versus 0.1% for cervical cancer, and 15.4% versus 14.3% for CIN2+. Repeated CIN1 was not associated with higher subsequent CIN3+ risk, supporting conservative, risk-based follow-up after CIN1 biopsy.

## 1. Introduction

Cervical cancer remains a major public health problem despite being largely preventable. It is the fourth most common cancer among women worldwide, with approximately 660,000 new cases and 350,000 deaths reported in 2022 [1]. In Norway, cervical cancer is less common than in many parts of the world, but it still represents an important cause of cancer morbidity and mortality [2].

Virtually all cervical cancers are caused by persistent infection with oncogenic human papillomavirus (HPV), most commonly HPV16 and HPV18 [3,4]. Cervical cancer can be prevented through two complementary strategies: primary prevention by HPV vaccination and secondary prevention through cervical screening [5,6]. The main purpose of cervical screening programmes is to detect and treat high-grade cervical precancer before progression to invasive cancer [7].

The Norwegian Cervical Screening Programme was implemented in 1995 as a national organised programme for women aged 25–69 years. For many years, screening was based on cytology alone. From 2005, HPV testing was gradually incorporated in the triage of women with atypical squamous cells of undetermined significance (ASC-US) and low-grade squamous intraepithelial lesion (LSIL) cytology [8,9,10]. Primary HPV screening for women aged 34–69 years was introduced in Norway in 2015 in four pilot counties through a randomised implementation in which women were allocated to either HPV testing every five years or cytology every three years. Based on the pilot results, Norwegian health authorities decided in 2017 to replace cytology with five-yearly primary HPV screening for women aged 34–69 years in these counties. From 2019, this strategy was progressively rolled out to additional counties using the same randomised implementation approach, and by the end of 2021, HPV-based screening had been implemented for all women aged 34–69 years. From July 2023, primary HPV screening was also extended to women aged 25–33 years, making HPV-based testing the primary screening strategy for all women aged 25–69 years in the Norwegian Cervical Screening Programme [8,11,12]. This transition has increased the proportion of women with positive screening tests and has led to more colposcopies and more histologically normal and low-grade lesions such as cervical intraepithelial neoplasia grade 1 (CIN1), consistent with the higher sensitivity of HPV testing for detecting HPV infection and earlier cervical disease than cytology-based screening [11,13].

CIN1 is the most common histological diagnosis after referral for colposcopy and is generally regarded as the histological manifestation of HPV infection, usually reflecting a productive infection with low risk of progression compared with true cervical precancer [14,15]. Accordingly, CIN1 is usually managed conservatively with repeat cytology and HPV testing, because most lesions regress and only a minority progress to more severe disease. At the same time, follow-up after low-grade histology remains clinically important, since a subset of women will subsequently be diagnosed with CIN2+, and unnecessary treatment should be avoided because excisional procedures may cause obstetric and gynaecological morbidity [16,17].

The evidence base for management after CIN1 biopsy has improved in recent years but still leaves important questions unanswered. In an earlier Norwegian post-colposcopy study of women with abnormal cytology and/or positive HPV mRNA testing followed by a normal or CIN1 biopsy, 72/520 women (13.8%) were subsequently diagnosed with CIN3+, including seven cervical cancers. Among women with CIN1 at the first biopsy, 28/231 (12.1%) developed CIN3+, including three cervical cancers, highlighting that a low-grade biopsy finding after abnormal screening does not eliminate clinically relevant future risk [18]. More recently, Baasland et al. reported nationwide data from the Norwegian Cervical Screening Programme showing a 5-year cumulative incidence of 12.7% for CIN3+ after incident CIN1. CIN3+ risk varied substantially according to index cytology and HPV status, reaching 18.9% after high-grade index cytology and 16.6% among women with normal or low-grade cytology but HPV type 16/18 positivity, compared with 8.7% for other HPV types and 4.3% for HPV-negative women. These findings support risk-based follow-up after CIN1, but the study was based on incident CIN1 and did not specifically address whether CIN1 diagnosed in two consecutive years identifies a subgroup with higher subsequent CIN3+ risk [16].

This question is clinically relevant because persistence of low-grade abnormalities is often interpreted as a marker of progression risk and may lead to intensified surveillance or excisional treatment in routine practice. However, recent Italian active-surveillance data on persistent LSIL/CIN1 suggest that progression to CIN3 is uncommon, occurs mainly within the first two years, and does not continue to accumulate with longer persistence, supporting the view that LSIL/CIN1 and high-grade lesions may be biologically distinct rather than obligatory steps in a linear continuum [19]. Further real-world data are therefore needed to clarify whether women with CIN1 in two consecutive years truly differ in long-term risk from women with CIN1 diagnosed in a single year.

The aim of the present study was first to describe temporal trends in cervical screening activity and related diagnostic outcomes in Northern Norway during 2011–2025, including ASC-US+ cytology, positive HPV tests, biopsy use, and CIN2+ detection. Second, we aimed to estimate the 5-year risk of CIN3+ after histologically confirmed CIN1 biopsy and to compare this risk in two clinically relevant settings: CIN1 diagnosed in a single calendar year and CIN1 diagnosed in two consecutive calendar years. Secondary outcomes included CIN2+ as a clinically relevant treatment-related endpoint and cervical cancer as the most severe outcome.

## 2. Materials and Methods

### 2.1. Study Design and Setting

This was a retrospective, population-based cohort study based on anonymised pathology data from the Department of Clinical Pathology, University Hospital of North Norway (UNN), Tromsø, Norway, covering the period from 1 January 2011 to 31 December 2025. UNN is the sole pathology provider for the counties of Troms and Finnmark, ensuring near-complete regional capture of cervical cytology, HPV testing, and cervical histopathology, including diagnostic biopsies and excisional specimens. The study had two aims. First, we described annual screening-related activity and key downstream outcomes, including annual screened woman-records and the proportions with ASC-US+ cytology, a positive HPV test, biopsy, and CIN2+ during 2011–2025 (Table 1). Second, we estimated the absolute 5-year risk of subsequent CIN3+ after histologically confirmed CIN1, with CIN2+ and cervical cancer assessed as secondary endpoints in two clinically relevant settings: (i) women with CIN1 diagnosed in a single calendar year and (ii) women with CIN1 diagnosed in two consecutive calendar years. Because exact biopsy dates were not available in the extracted dataset, the two-consecutive-year cohort was defined by calendar year rather than by exact biopsy-to-biopsy intervals, and the median interval between the first and second CIN1 biopsy could not be calculated. In Norwegian clinical practice, women with a normal or low-grade cervical biopsy after an abnormal screening test are generally managed conservatively with repeat cytology and HPV testing after approximately 12 months rather than immediate treatment, making biopsy-based risk estimates highly relevant for clinical follow-up [18].

### 2.2. Data Source and Study Population

All cervical cytology, HPV, and histopathology records were extracted from the laboratory information system SymPathy v5.14.4.2 (Tietoevry, Espoo, Finland), which is routinely used at the Department of Clinical Pathology, UNN. Records were linked at the individual level using a unique patient identifier available within the anonymised dataset.

For the descriptive analyses in Table 1, we created annual datasets covering 2011 through 2025 to describe temporal changes in screening-related activity and downstream diagnostic outcomes. The denominator for each year was the number of unique screened women with at least one cervical sample recorded in that calendar year, including cytology and/or HPV testing from screening, follow-up, or symptom-based examinations. Within each year, we then identified women with ASC-US+ cytology, women with a positive HPV test, women who underwent biopsy, and women diagnosed with CIN2+. ASC-US+ was defined as abnormal cytology including ASC-US, LSIL, ASC-H, AGC, and HSIL. Positive HPV tests were defined as registered positive HPV results in the pathology dataset. Biopsy was defined as at least one cervical histology specimen recorded in the same calendar year, and CIN2+ was defined histologically as CIN2, CIN3, adenocarcinoma in situ (ACIS), squamous cell carcinoma (SCC), or adenocarcinoma of the cervix (ACC). To avoid multiple counting within the same year, duplicate records were removed by retaining one record per woman and calendar year for each descriptive analysis. These annual descriptive outcomes were calendar-year-based measures and were not necessarily linked to the same screening episode.

Positive HPV tests were defined as registered positive HPV results in the pathology dataset. HPV testing methods changed during the study period. In 2011, the 5-type HPV mRNA assay PreTect HPV-Proofer (PreTect AS, Klokkarstua, Norway), detecting E6/E7 mRNA from HPV types 16, 18, 31, 33, and 45, was used for triage of ASC-US/LSIL. From 2012, the 14-type cobas HPV test (Roche Diagnostics, Basel, Switzerland) was used on the cobas 4800 platform for triage of ASC-US/LSIL. From 2016 to 2020, cytology samples were routinely co-tested with the 3-type HPV mRNA assay PreTect SEE (PreTect AS, Klokkarstua, Norway), targeting HPV types 16, 18, and 45. From 2019, primary HPV DNA screening with the cobas HPV test (Roche Diagnostics, Basel, Switzerland) was implemented for women aged 34–69 years. In 2025, Roche cobas testing was transferred from the cobas 4800 platform to the cobas 6800 platform; this affected only the annual descriptive data, as the CIN1 risk cohorts were restricted to index CIN1 biopsies from 2011 to 2020. The cobas HPV test reports HPV type 16 and HPV type 18 separately, while the remaining included HPV types are reported as a pooled ‘other types’ result. Extended HPV genotyping was not available in the dataset.

For the risk analyses, the source histology file comprised all cervical histological diagnoses of CIN1, CIN2, CIN3, ACIS, SCC, and ACC recorded during the study period. Available variables included patient identifier, age, year of diagnosis, pathology request number, topography code, diagnostic code, cleaned diagnostic code, and grouped diagnosis category. Histological diagnoses reflected routine diagnostic reporting at the Department of Clinical Pathology, UNN, and no additional expert review was performed specifically for this study. Routine cervical histopathology assessment was stable during the study period and was performed by a relatively stable group of experienced pathologists. Most cervical biopsy diagnoses were based on haematoxylin and eosin sections alone. In routine practice, all high-grade cervical lesions, including CIN2, CIN3, ACIS, SCC, and ACC, are evaluated by two independent experienced pathologists. Ambiguous lesions at the LSIL/CIN1 versus HSIL/CIN2 threshold were adjudicated by the reporting pathologist, with p16 immunohistochemistry using CINtec Histology (Roche Diagnostics, Basel, Switzerland) when indicated to support this distinction. The laboratory did not use p16/Ki-67 dual-staining immunohistochemistry for cervical biopsy histology. From 2019 onwards, the EagleEye AI system (EagleEye, Oslo, Norway), subsequently evaluated as a decision-support tool for cervical biopsy diagnostics at the CIN2+ threshold [20], was available as an adjunctive decision-support and quality-assurance tool for cervical biopsies, intended to help identify possible high-grade foci; final diagnoses remained the responsibility of the pathologist, and diagnostic criteria were not changed.

For the single-calendar-year CIN1 analysis, we identified all women with histologically confirmed CIN1 (diagnostic code M74006) in each calendar year from 2011 through 2020. Separate annual CIN1 cohorts were created for 2011, 2012, 2013, 2014, 2015, 2016, 2017, 2018, 2019, and 2020. Within each annual cohort, duplicate records were removed by patient identifier, retaining the first CIN1 record for each woman in the given year. Women were not excluded because of prior CIN2+, prior excisional treatment, or earlier abnormal screening history before the index CIN1 biopsy. The cohort should therefore be interpreted as a real-world population of women with a recorded CIN1 biopsy in routine pathology practice, rather than as a strictly incident CIN1 cohort.

For the two-consecutive-year CIN1 analysis, overlapping two-year cohorts were created: 2011–2012, 2012–2013, 2013–2014, 2014–2015, 2015–2016, 2016–2017, 2017–2018, 2018–2019, and 2019–2020. Women were included in a given two-year cohort only if they had at least one CIN1 biopsy in both calendar years. After this selection, each two-year cohort was reduced to one record per woman by patient identifier, retaining the record from year 2, which served as the index record for subsequent follow-up. As in the single-year analysis, women were not excluded because of prior CIN2+, prior treatment, or earlier abnormal screening history. This design was chosen to estimate the observed 5-year risk after repeated CIN1 in routine clinical practice, with follow-up starting only after confirmation of CIN1 in two consecutive years.

### 2.3. Definition of Follow-Up and Outcomes

Follow-up was based on subsequent histological diagnoses recorded in the pathology dataset, including both diagnostic cervical biopsies and excisional specimens. For the single-year analysis, women with CIN1 in a given index year were followed for CIN3+ during the subsequent five calendar years: 2011 CIN1 was linked to outcomes in 2012–2016, 2012 CIN1 to outcomes in 2013–2017, and so forth through 2020 CIN1 linked to outcomes in 2021–2025. For the two-consecutive-year analysis, follow-up started after year 2 of the CIN1 pair: women with CIN1 in 2011–2012 were linked to outcomes in 2013–2017, women with CIN1 in 2012–2013 to outcomes in 2014–2018, and so forth through 2019–2020 linked to outcomes in 2021–2025.

The primary endpoint was histologically confirmed CIN3+, defined as CIN3, adenocarcinoma in situ (ACIS), squamous cell carcinoma (SCC), or adenocarcinoma of the cervix (ACC). Secondary endpoints were CIN2+, defined as CIN2, CIN3, ACIS, SCC, or ACC, and cervical cancer, defined as SCC or ACC. This endpoint hierarchy emphasizes CIN3+ as the more specific premalignant endpoint, while retaining CIN2+ as a clinically relevant treatment-related outcome in routine management. This is consistent with prior Norwegian studies evaluating progression after low-grade cervical histology, where CIN2+ has been used as a main treatment-relevant outcome and CIN3+ as a more specific premalignant endpoint [16,18].

In Norway, histologically confirmed CIN1 is generally managed conservatively and is not routinely treated by excision. Excisional treatment with LEEP/LLETZ is primarily recommended for histologically confirmed high-grade lesions, such as CIN2 or CIN3, and is used as treatment rather than as routine diagnostic work-up. Diagnostic excision may be considered in selected cases, for example when high-grade cytology persists despite normal or CIN1 biopsy findings, or when invasive disease cannot be excluded, but this is not routine management for CIN1.

Women were not excluded or censored because of subsequent excisional treatment. The denominator for each 5-year risk estimate was defined by the index CIN1 cohort. If CIN3+ was diagnosed in a subsequent biopsy or excisional specimen during the relevant 5-year follow-up window, the woman was counted as having reached the primary endpoint. If a subsequent excisional specimen showed normal histology, CIN1, or CIN2 only, the woman remained in the denominator and was classified as not having CIN3+ unless a later CIN3+ diagnosis was recorded within the same follow-up window.

Secondary endpoints were assessed in the same way. Thus, women with CIN2, CIN3, ACIS, SCC, or ACC during follow-up were counted as having reached the CIN2+ endpoint, whereas women with SCC or ACC were counted as having reached the cervical cancer endpoint.

If a woman had more than one abnormal histological diagnosis within a five-year follow-up window, only the most severe diagnosis was retained. For each follow-up window, women were therefore represented by a single outcome record corresponding to their highest-grade diagnosis. Women without a histological diagnosis meeting the relevant endpoint definition during the five-year window were classified as non-cases for that endpoint.

### 2.4. Data Management and Record Linkage

All datasets were handled stepwise in IBM SPSS Statistics, version 29.0 (IBM Corp., Armonk, NY, USA). For each follow-up window, outcome files were first reduced to one record per woman by patient identifier, retaining the record with the highest-ranked histological diagnosis. The annual or two-year CIN1 cohorts were then linked to the corresponding follow-up outcome file by patient identifier using left joins, thereby preserving all women in the CIN1 cohorts regardless of whether a subsequent CIN3+, CIN2+, or cervical cancer diagnosis was observed.

For the primary endpoint, women were classified as CIN3+ cases if CIN3, ACIS, SCC, or ACC was recorded in a subsequent biopsy or excisional specimen during the relevant five-year follow-up window. For secondary endpoints, the same linkage procedure was applied to identify CIN2+ and cervical cancer outcomes. Thus, each woman contributed one outcome record per follow-up window, corresponding to the highest-grade histological diagnosis observed during follow-up.

### 2.5. Episode-Based and Person-Based Analyses

Two complementary analytical approaches were used. First, an episode-based approach was applied, in which each eligible single-calendar-year CIN1 cohort or each eligible two-consecutive-calendar-year CIN1 cohort contributed separately to the analysis. In this approach, the same woman could contribute more than once across calendar years or overlapping two-year windows if she fulfilled the inclusion criteria repeatedly.

Second, a person-based approach was adopted to obtain estimates based on unique women only. For this analysis, all eligible cohorts within each design were merged into one combined dataset and then deduplicated by patient identifier, retaining the first eligible CIN1 record for each woman. This provided one observation per woman across the entire study period and allowed assessment of whether repeated inclusion of the same woman materially influenced the estimated risk. The rationale for examining both episode-based and person-based estimates was the same as in other Norwegian studies of cervical follow-up, namely to distinguish between risk per eligible episode and risk per woman.

### 2.6. Statistical Analysis

The primary measure was the absolute 5-year risk of CIN3+. Secondary measures were the absolute 5-year risks of CIN2+ and cervical cancer, reported as the number of events divided by the number of women at risk (*n*/*N*, %) with 95% confidence intervals (CIs). Risks were calculated separately for each single-calendar-year CIN1 cohort and each two-consecutive-calendar-year CIN1 cohort and then summarised across all eligible cohorts. Descriptive frequency analyses were used to estimate the proportions of women developing CIN2+, CIN3+, and cervical cancer during follow-up. In the combined analyses, both episode-based and person-based estimates were reported. Ninety-five percent confidence intervals for absolute risks were calculated using binomial exact methods. Temporal trends in annual proportions of screening-related outcomes were assessed using the Cochran–Armitage test for trend. Because ASC-US+ and CIN2+ showed non-monotonic patterns over time, these outcomes were primarily described descriptively rather than summarised by a single linear trend estimate across the full study period. Statistical analyses were performed using IBM SPSS Statistics, version 29.0 (IBM Corp., Armonk, NY, USA).

### 2.7. Ethics

The study was based on retrospective analysis of de-identified routine pathology data and involved no patient contact or intervention. The project was assessed by the Regional Committee for Medical and Health Research Ethics, REK Nord, as a quality-assurance project and was therefore considered outside the scope of the Norwegian Health Research Act. Ethical review and approval were waived for this study (application no. 986556; assessment date: 17 April 2026). Individual informed consent was not required.

## 3. Results

**Figure 1:**
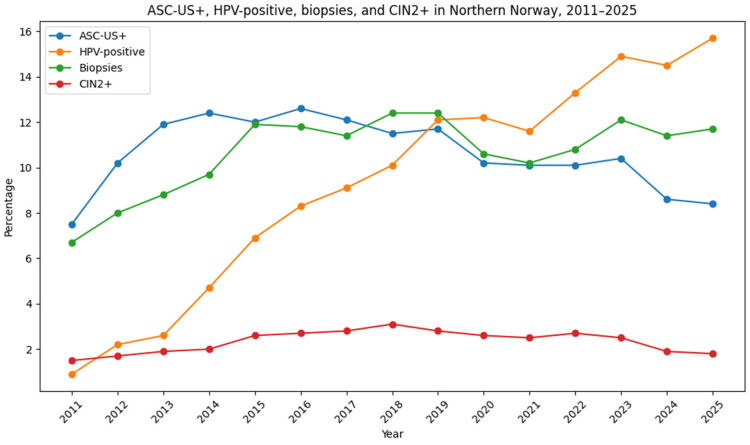
Annual proportions of women with ASC-US+ cytology, positive HPV test, biopsy, and CIN2+ among screened woman-records in Northern Norway, 2011–2025. The proportion of women with a positive HPV test increased steadily over time, while the proportion with ASC-US+ peaked in the mid-study period and then declined. Biopsy rates increased during the first part of the study period and remained relatively stable thereafter. The proportion diagnosed with CIN2+ rose until 2018 and declined in the most recent years. Abbreviations: ASC-US+, atypical squamous cells of undetermined significance or worse; HPV, human papillomavirus; CIN2+, cervical intraepithelial neoplasia grade 2 or worse.

Across 2011–2025, the annual datasets comprised 334,471 screened woman-records. Overall, 35,796 women had ASC-US+ cytology (10.7%), 29,723 had a positive HPV test (8.9%), 35,416 underwent biopsy (10.6%), and 7870 were diagnosed with CIN2+ (2.4%) (Table 1). The annual number of screened women declined over time, from 25,595 in 2011 to 17,388 in 2025. The proportion of women with a positive HPV test increased steadily throughout the study period, from 0.9% in 2011 to 15.7% in 2025 (*p* for trend < 0.001). The proportion undergoing biopsy also increased during the first part of the study period, from 6.7% in 2011 to 12.4% in 2018–2019, and then remained relatively stable through 2025. In contrast, ASC-US+ and CIN2+ showed non-linear temporal patterns: ASC-US+ increased from 7.5% in 2011 to a peak of 12.6% in 2016 before declining to 8.4% in 2025, while CIN2+ rose from 1.5% in 2011 to 3.1% in 2018 and then declined to 1.8% in 2025 (Table 1, Figure 1).

Overall, these data show substantial temporal changes in screening outcomes. HPV positivity increased steadily over time and can be summarised statistically by a significant Cochran–Armitage test for trend (*p* for trend < 0.001). By contrast, ASC-US+, biopsy rates, and CIN2+ showed non-monotonic patterns over time and are therefore more appropriately described descriptively than summarised by a single linear trend estimate across the full study period. Figure 1 illustrates these divergent temporal patterns, with steadily increasing HPV positivity, biopsy rates that increased during the first part of the study period and then remained relatively stable, and CIN2+ detection that rose until 2018 before declining in the most recent years.

A total of 6780 women with histologically confirmed CIN1 diagnosed in a single calendar year between 2011 and 2020 were included in the annual cohort analyses. During the subsequent 5-year follow-up, 1010 women developed CIN2+, corresponding to an overall risk of 14.9% (95% CI 14.1–15.8). Of these, 275 women developed CIN3+ (4.1%; 95% CI 3.6–4.6), and 14 developed cervical cancer (0.2%; 95% CI 0.1–0.3) (Table 2).

The 5-year risk of CIN2+ was relatively stable across calendar years, ranging from 12.8% in the 2013 cohort to 16.9% in the 2019 cohort. The corresponding risk of CIN3+ ranged from 2.8% in the 2011 cohort to 5.4% in the 2012 cohort. Cervical cancer was rare in all annual cohorts, with no cases observed after CIN1 diagnosed in 2011 or 2012 and only 1–3 cases in the remaining cohorts.

Overall, these findings indicate that the risk of subsequent CIN2+ after a CIN1 biopsy was moderate and relatively stable across calendar-year cohorts, whereas the risk of CIN3+ was substantially lower and the risk of cervical cancer remained very low throughout the study period.

When all annual CIN1 cohorts were combined, some women contributed to more than one cohort because CIN1 could be diagnosed in more than one calendar year. Before restructuring, the pooled dataset therefore included 6780 records, with a 5-year risk of 14.9% for CIN2+ (95% CI 14.1–15.8), 4.1% for CIN3+ (95% CI 3.6–4.6), and 0.2% for cervical cancer (95% CI 0.1–0.3) (Table 3).

After restructuring to unique women by retaining only the first CIN1 record for each woman across the full study period, the dataset was reduced to 5286 women. In this person-based analysis, 815 women developed CIN2+ during follow-up, corresponding to a 5-year risk of 15.4% (815/5286; 95% CI 14.5–16.4). The corresponding 5-year risks were 4.3% for CIN3+ (227/5286; 95% CI 3.8–4.9) and 0.2% for cervical cancer (10/5286; 95% CI 0.1–0.3) (Table 3).

Thus, restricting the analysis to unique women had only a minor impact on the estimated risks. The absolute risk increased by 0.5 percentage points for CIN2+ and by 0.2 percentage points for CIN3+, whereas the risk of cervical cancer remained unchanged. This suggests that the overall findings were robust and were not materially influenced by repeated inclusion of the same woman in different annual CIN1 cohorts.

Before restructuring, women could contribute to more than one annual CIN1 cohort; after restructuring, only the first CIN1 record per woman was retained across the full study period.

We next evaluated women with histologically confirmed CIN1 in two consecutive calendar years and assessed their 5-year risk of subsequent CIN2+ starting after year 2. Before restructuring, the analysis of all overlapping two-year windows included 836 records. In this episode-based analysis, 113 women developed CIN2+, corresponding to a 5-year risk of 13.5% (113/836; 95% CI 11.4–16.0). The corresponding 5-year risks were 3.1% for CIN3+ (26/836; 95% CI 2.1–4.5) and 0.1% for cervical cancer (1/836; 95% CI 0.0–0.7) (Table 4).

After restructuring to unique women across the full study period, 697 women remained in the cohort. In this person-based analysis, 100 women developed CIN2+, yielding a 5-year risk of 14.3% (100/697; 95% CI 11.9–17.1). The corresponding 5-year risks were 3.4% for CIN3+ (24/697; 95% CI 2.3–5.1) and 0.1% for cervical cancer (1/697; 95% CI 0.0–0.8) (Table 4).

As in the single-year analysis, restructuring to unique women resulted in only small changes in the estimated risks. The risk of CIN2+ increased modestly from 13.5% to 14.3%, and the risk of CIN3+ from 3.1% to 3.4%, whereas the risk of cervical cancer remained unchanged. These findings indicate that repeated inclusion of the same woman in overlapping two-year CIN1 windows had little impact on the overall risk estimates.

In the person-based comparison, women with CIN1 diagnosed in a single calendar year had a 5-year risk of 15.4% for CIN2+ (815/5286; 95% CI 14.5–16.4), 4.3% for CIN3+ (227/5286; 95% CI 3.8–4.9), and 0.2% for cervical cancer (10/5286; 95% CI 0.1–0.3). Among women with CIN1 diagnosed in two consecutive calendar years, the corresponding 5-year risks were 14.3% for CIN2+ (100/697; 95% CI 11.9–17.1), 3.4% for CIN3+ (24/697; 95% CI 2.3–5.1), and 0.1% for cervical cancer (1/697; 95% CI 0.0–0.8) (Table 5).

Thus, CIN1 diagnosed in two consecutive calendar years was not associated with a higher subsequent 5-year risk than CIN1 diagnosed in a single calendar year. The observed risks of both CIN2+ and CIN3+ were slightly lower in the two-consecutive-year cohort, while cervical cancer remained rare in both groups. These findings suggest that repeated CIN1 diagnoses over two consecutive years did not identify a subgroup with clearly increased risk of progression during the following five years.

## 4. Discussion

### 4.1. Principal Findings

Our study has two main findings. First, the descriptive analyses showed substantial changes in cervical screening activity and downstream diagnostic outcomes in Northern Norway during 2011–2025, with increasing HPV positivity, changing biopsy activity, and a recent decline in CIN2+ detection.

Second, in the biopsy-based risk analyses, the 5-year risk of CIN3+ after CIN1 was low, and the risk of cervical cancer was very low. CIN2+ risk was higher, as expected, but CIN2 is a less specific endpoint with more variable biological potential. Importantly, CIN1 diagnosed in two consecutive calendar years did not identify a subgroup with higher subsequent CIN3+ risk than CIN1 diagnosed in a single calendar year. These findings support conservative, risk-based follow-up after CIN1 biopsy.

### 4.2. Temporal Trends in Screening Activity and Diagnostic Outcomes

The temporal increase in registered HPV-positive results in Norway likely reflects major technological and programme-related changes in the Norwegian Cervical Screening Programme, particularly the expanded use of HPV testing in triage and the phased introduction of primary HPV screening [8,11]. In the present study, HPV positivity refers to any registered positive HPV test result in the pathology dataset and was not based on a single assay throughout the study period. During the earlier years, HPV testing was used mainly for triage of women with ASC-US or LSIL. From 2019, primary HPV screening with the cobas HPV test was gradually implemented for women aged 34–69 years and was completed nationally by 2022. From July 2023, this strategy was extended to women aged 25–33 years [8,11,12]. This interpretation is supported by Deen et al., who reported a marked increase in colposcopy referrals and cervical biopsy workload after implementation of HPV triage. The diagnostic profile shifted towards more low-grade findings, particularly HPV-related changes and CIN1, with a lower mean CIN score, indicating that HPV-based triage increases detection of low-grade lesions and has implications for colposcopy and histopathology resources [21].

In addition, HPV testing methods changed over time. The shift from a 5-type HPV mRNA assay to a 14-type HPV DNA assay, together with the temporary use of an additional 3-type HPV mRNA test, may have increased registered HPV positivity and led to closer follow-up of some women compared with earlier practice [22,23]. The observed increase in Table 1 and Figure 1 should therefore be interpreted as an overall increase in registered HPV positivity in routine practice, influenced by changes in screening policy, test indication, assay type, and target population, rather than as an increase measured by one unchanged HPV assay.

The pattern for ASC-US+ is also consistent with the change in screening strategy. The proportion of ASC-US+ increased during the cytology-based and transitional years but declined after implementation of primary HPV screening. A plausible explanation is that women with a negative primary HPV test are classified as screen-negative and do not undergo reflex cytology, thereby reducing the opportunity to detect cytological abnormalities in the overall screened population [11,12,24]. In parallel, the increase in biopsy activity was likely driven primarily by the larger number of women with positive HPV screening results and by more intensive follow-up algorithms under HPV-based screening. This occurred despite a longer screening interval, as the programme shifted from cytology every three years to HPV testing every five years. Thus, the rise in biopsy rates likely reflects both increased registered HPV positivity and changes in referral and surveillance patterns during the transition to HPV-based screening [11,12,24].

The transition to primary HPV screening may also have changed the underlying risk profile of women diagnosed with CIN1. Compared with cytology-based triage, primary HPV screening is more sensitive and may identify women with HPV infection before cytological abnormalities or histologically confirmed high-grade lesions have developed. This can increase referral to follow-up and biopsy and may lead to more histologically normal and low-grade biopsy findings.

Consequently, the CIN1 population in the HPV-screening era may include a larger proportion of women with transient HPV infections or early low-grade lesions that would not have progressed to CIN3+. This may dilute the observed progression-risk pool compared with older cytology-based cohorts, in which biopsy referral was more often triggered by established cytological abnormalities. Earlier detection through HPV testing may also introduce lead time, potentially lowering the observed risk of CIN3+ within a fixed five-year follow-up window.

### 4.3. Explaining the Decline in CIN2+ Detection

The decline in CIN2+ detection after 2018 is particularly noteworthy. Several factors may have contributed. One plausible explanation is that women with a negative 3-type HPV mRNA result had a very low subsequent risk of CIN2+/CIN3+, while the use of this assay also enabled earlier detection of high-grade lesions among some women with normal cytology, potentially leaving fewer CIN2+ lesions to be detected at the next screening round [23]. In addition, from 2022 onwards, the first routinely HPV-vaccinated birth cohorts entered the screening programme at age 25 years in Norway, which would be expected to reduce the occurrence of HPV16/18-associated high-grade cervical disease [25]. A further contributing factor may be that women with a negative primary HPV test in 2019 returned after five rather than three years, and these women represent a lower-risk group [12]. Taken together, these factors likely contributed to the decline in calendar-year-based CIN2+ detection observed in the most recent years despite continuing increases in HPV positivity.

### 4.4. Clinical Interpretation of Repeated CIN1

The main clinical finding of the present study is that repeated CIN1 in two consecutive calendar years did not identify a subgroup with clearly higher long-term risk than CIN1 diagnosed in a single calendar year. Although persistence of low-grade abnormalities is often assumed to indicate increased progression risk, this assumption should be interpreted cautiously when applied to histologically confirmed CIN1 [14,19]. Repeated CIN1 may partly reflect persistent low-grade HPV-related changes rather than imminent progression to high-grade disease, which is consistent with guideline-based preference for observation, the low subsequent risk reported for persistent histological CIN1, and the possibility that CIN1 and CIN3 may coexist as biologically distinct lesions rather than represent obligatory sequential steps [14,19].

This interpretation is also supported by the biology of HPV-associated cervical lesions. CIN1 usually reflects a productive HPV infection in which the viral genome is maintained predominantly in episomal form, whereas CIN3 is more often characterised by deregulated viral oncogene expression and may be associated with integration of HPV DNA into the host genome. However, HPV integration is not universal in CIN3, and progression is also influenced by persistent deregulated E6/E7 expression and accumulation of host-cell genetic alterations [26].

### 4.5. Implications for Clinical Management

The interpretation of CIN2+ as a treatment-relevant endpoint should also take into account the heterogeneous nature and substantial spontaneous regression rate of CIN2, particularly in younger women. CIN2 is less reproducible than CIN3 and represents a biologically mixed category, including both transient HPV-associated lesions and true precancerous lesions. Several studies have shown that a large proportion of CIN2 lesions regress under active surveillance. In a systematic review and meta-analysis, approximately half of untreated CIN2 lesions regressed during follow-up, with higher regression rates among women younger than 30 years [27]. Earlier studies also support the heterogeneous and frequently regressive nature of CIN2; Castle et al. estimated that approximately 40% of CIN2 lesions may regress over 2 years [28], while Moscicki et al. reported regression of CIN2 in adolescents and young women in 63% by 2 years and 68% by 3 years [29]. More recent population-based and prospective cohort studies have similarly supported active surveillance for selected women with CIN2, especially younger women, while identifying persistent HPV16/18 infection, high-grade cytology, and larger lesions as markers of increased progression risk [30,31].

These findings support conservative management after CIN1 biopsy and do not support excisional treatment based solely on repeated histological CIN1 findings. Our data are therefore more consistent with a risk-based follow-up strategy than with intervention triggered solely by CIN1 diagnosed in consecutive calendar years. This interpretation is compatible with prior Norwegian and international literature showing that low-grade biopsy findings do not eliminate future high-grade risk, including CIN3+, but that repeated low-grade histology does not necessarily translate into progressively increasing long-term CIN3+ risk [14,16,18]. Importantly, repeated histological CIN1 should not be equated with persistent HPV detection. The present study could not evaluate duration of HPV positivity, genotype-specific HPV persistence, or individual vaccination status, and the findings should therefore not be interpreted as evidence against the clinical importance of persistent HPV detection. In this context, our study adds real-world evidence from a population-based regional cohort showing that CIN1 diagnosed in two consecutive calendar years did not identify a subgroup with higher subsequent CIN3+ risk.

### 4.6. Strengths and Limitations

An important strength of this study is the large population-based dataset from a defined geographic region. The Department of Clinical Pathology at UNN is the sole pathology provider for Troms and Finnmark and receives cervical screening samples, follow-up samples, cervical biopsies, excisional specimens, and hysterectomy specimens from women in this region, ensuring near-complete regional capture of cervical pathology. This reduces loss of follow-up within the study area and supports internal consistency of the data.

In addition, cervical histopathology was reported by a relatively stable group of experienced pathologists over time, which likely contributed to diagnostic consistency across the study period. Routine histological assessment was stable, with most cervical biopsy diagnoses based on haematoxylin and eosin sections alone. The laboratory did not use p16/Ki-67 dual-staining immunohistochemistry for cervical biopsy histology. p16 immunohistochemistry was used only in selected difficult cases to support the distinction between LSIL/CIN1 and HSIL/CIN2, and all high-grade cervical lesions were evaluated by two independent experienced pathologists. From 2019 onwards, EagleEye AI was available as an adjunctive decision-support and quality-assurance tool [20], but final diagnoses remained the responsibility of the pathologist and diagnostic criteria were not changed.

The two complementary analytical designs, together with both episode-based and person-based analyses, also strengthen the interpretation. The very small differences after restructuring indicate that the findings were robust and not materially driven by repeated inclusion of the same woman.

The study also has limitations. First, exact biopsy dates were not available in the dataset used for the present analysis. The two-consecutive-year CIN1 cohort was therefore defined by calendar year rather than by exact biopsy-to-biopsy intervals, and the median interval between the first and second CIN1 biopsy could not be calculated. Accordingly, CIN1 diagnosed in two consecutive calendar years should be interpreted as a pragmatic clinical proxy for repeated CIN1 during follow-up, rather than as a precise measure of biological persistence. In Norwegian clinical practice, women with a normal or CIN1 cervical biopsy after an abnormal screening result are generally followed with repeat cytology and HPV testing after approximately 12 months. Thus, most repeated CIN1 biopsies in consecutive calendar years are likely to reflect routine follow-up, but the actual interval between biopsies may have varied between individual women. In addition, neither HPV persistence nor CIN1 persistence has a strict biological cut-off, because HPV infections and low-grade lesions may persist for several years before spontaneous clearance or regression.

Second, women with prior CIN2+, prior excisional treatment, or earlier abnormal screening history were not excluded before the index CIN1 biopsy. The study therefore does not estimate the natural history of incident CIN1 in women without previous abnormalities. Rather, the results reflect the observed 5-year risk after a recorded CIN1 biopsy in routine pathology practice, including women with variable prior screening and treatment histories. Prior CIN2+ or treatment may modify subsequent risk and could not be accounted for in the present analysis.

Third, the observed temporal trends in HPV positivity, biopsy use, and CIN2+ detection were strongly influenced by changes in screening policy, HPV testing methods, and vaccinated cohort entry, which limits direct comparison across calendar years. In addition, the annual descriptive measures in Table 1 and Figure 1 were calendar-year-based and should not be interpreted as directly episode-linked outcomes following a specific screening test in the same year.

Fourth, the main risk estimates were not adjusted for age, HPV genotype, vaccination status, or index cytology, although these factors may modify the risk of CIN3+ after CIN1. HPV results were unavailable for many women, particularly before the introduction of primary HPV screening in 2019, and extended HPV genotyping was not available in the dataset. HPV testing methods also changed during the study period. The Roche cobas HPV DNA assay provided partial genotyping only, with separate reporting of HPV type 16 and HPV type 18, while the remaining included HPV types were reported as a pooled ‘other types’ result. The assay did not provide separate reporting of HPV type 45, HPV 18/45, or aggregate genotype groups such as HPV 31/33/52/58 or HPV 35/39/51/56/59/66/68. Therefore, the study could not assess genotype-specific progression risk after CIN1, including the potential contribution of individual HPV types within the pooled ‘other types’ category. Individual HPV vaccination status was not available. However, national school-based HPV vaccination in Norway started in 2009, targeting 12-year-old girls, with girls born in 1997 representing the first vaccine-eligible birth cohort [32]. Since CervicalScreen Norway invites women aged 25–69 years, this first routinely vaccinated cohort reached screening age in 2022 [8]. The women included with CIN1 biopsies during 2011–2020 are therefore unlikely to have been substantially influenced by routine childhood vaccination. Index cytology was not included in the main risk models because Norwegian guidelines recommend 12-month follow-up after abnormal screening results followed by a normal or CIN1 biopsy, regardless of index cytology. The reported risks should therefore be interpreted as overall population-based risks within this guideline-based follow-up pathway, rather than as individualized or genotype-specific risk estimates.

Fifth, CIN3+ was used as the primary endpoint because it is a more specific and clinically meaningful marker of cervical precancer than CIN2+, which is less reproducible and more likely to regress [33,34]. However, CIN2+ was retained as a secondary endpoint because CIN2 remains clinically relevant in routine management and is often used as a treatment-related outcome. CIN2 is a heterogeneous diagnostic category and may include lesions with variable malignant potential, particularly in younger women [27,28,29,30,31].

Finally, because this was a retrospective pathology-based study, the results reflect real-world clinical practice rather than a standardized prospective follow-up protocol.

### 4.7. Implications for Future Risk Stratification and Research

Overall, the present findings suggest that histologically confirmed CIN1 should continue to be managed conservatively, including when CIN1 is diagnosed in two consecutive calendar years. In an era of increasing HPV positivity and changing referral patterns under primary HPV screening, these results are clinically relevant because they argue against overtreatment of women with repeated low-grade histology and support further refinement of follow-up using additional risk markers such as age, index cytology, HPV genotype, and vaccination status [35].

## 5. Conclusions

In conclusion, the 5-year risk of CIN3+ after histologically confirmed CIN1 biopsy was low, and the risk of cervical cancer was very low. CIN2+ risk was higher, but CIN2 is a less specific endpoint with more variable biological potential. Women with CIN1 diagnosed in two consecutive calendar years did not have a higher subsequent 5-year risk of CIN3+ than women with CIN1 diagnosed in a single calendar year. These findings support conservative, risk-based follow-up after CIN1 biopsy and do not support excisional treatment based solely on repeated CIN1. In the era of primary HPV screening, increasing HPV positivity, and entry of vaccinated cohorts into screening, future studies with access to detailed HPV genotype data, duration of HPV positivity, index cytology, age, and individual vaccination status may further refine risk stratification and management after CIN1.

## Figures and Tables

**Table 1:** Annual screened woman-records and proportions with ASC-US+ cytology, positive HPV test, biopsy, and CIN2+ in Northern Norway, 2011–2025, based on calendar-year datasets.

Year	Screened Women, *n*	ASC-US+, *n* (%)	HPV-Positive, *n* (%)	Biopsies, *n* (%)	CIN2+, *n* (%)
2011	25,595	1920 (7.5)	239 (0.9)	1722 (6.7)	388 (1.5)
2012	22,466	2285 (10.2)	489 (2.2)	1800 (8.0)	388 (1.7)
2013	23,776	2838 (11.9)	609 (2.6)	2097 (8.8)	448 (1.9)
2014	24,822	3076 (12.4)	1158 (4.7)	2417 (9.7)	500 (2.0)
2015	22,937	2746 (12.0)	1573 (6.9)	2738 (11.9)	598 (2.6)
2016	22,633	2861 (12.6)	1880 (8.3)	2671 (11.8)	602 (2.7)
2017	25,055	3033 (12.1)	2268 (9.1)	2865 (11.4)	705 (2.8)
2018	23,320	2677 (11.5)	2352 (10.1)	2899 (12.4)	730 (3.1)
2019	22,033	2567 (11.7)	2676 (12.1)	2726 (12.4)	626 (2.8)
2020	21,428	2180 (10.2)	2621 (12.2)	2264 (10.6)	550 (2.6)
2021	24,395	2455 (10.1)	2822 (11.6)	2498 (10.2)	620 (2.5)
2022	21,129	2125 (10.1)	2806 (13.3)	2284 (10.8)	568 (2.7)
2023	19,429	2021 (10.4)	2893 (14.9)	2343 (12.1)	490 (2.5)
2024	18,065	1549 (8.6)	2613 (14.5)	2066 (11.4)	339 (1.9)
2025	17,388	1463 (8.4)	2724 (15.7)	2026 (11.7)	318 (1.8)
Total	334,471	35,796 (10.7)	29,723 (8.9)	35,416 (10.6)	7870 (2.4)

Abbreviations: ASC-US+, atypical squamous cells of undetermined significance or worse; HPV, human papillomavirus; CIN2+, cervical intraepithelial neoplasia grade 2 or worse. Percentages are calculated using the annual number of screened woman-records as the denominator. Biopsies refer to women who underwent at least one cervical biopsy during the same calendar year. CIN2+ refers to women diagnosed with CIN2+ during the same calendar year. The data are based on annual calendar-year datasets and represent woman-records rather than unique women across the full study period.

**Table 2:** Five-year risk of CIN2+, CIN3+, and cervical cancer after CIN1 diagnosed in a single calendar year.

Index Year of CIN1	Follow-Up Period	*N*	CIN2+, *n* (%)	CIN3+, *n* (%)	Cancer, *n* (%)
2011	2012–2016	217	34 (15.7)	6 (2.8)	0 (0.0)
2012	2013–2017	314	50 (15.9)	17 (5.4)	0 (0.0)
2013	2014–2018	439	56 (12.8)	14 (3.2)	2 (0.5)
2014	2015–2019	619	86 (13.9)	23 (3.7)	2 (0.3)
2015	2016–2020	851	121 (14.2)	34 (4.0)	1 (0.1)
2016	2017–2021	877	127 (14.5)	31 (3.5)	0 (0.0)
2017	2018–2022	925	125 (13.5)	40 (4.3)	2 (0.2)
2018	2019–2023	906	138 (15.2)	37 (4.1)	2 (0.2)
2019	2020–2024	878	148 (16.9)	45 (5.1)	2 (0.2)
2020	2021–2025	754	125 (16.6)	28 (3.7)	3 (0.4)
Total	2011–2025	6780	1010 (14.9)	275 (4.1)	14 (0.2)

Abbreviations: CIN1, cervical intraepithelial neoplasia grade 1; CIN2+, cervical intraepithelial neoplasia grade 2 or worse; CIN3+, cervical intraepithelial neoplasia grade 3 or worse. The index year denotes the calendar year in which CIN1 was first diagnosed, and outcomes were assessed during the subsequent 5-year follow-up period. Percentages are calculated using the number of women in each annual CIN1 cohort as the denominator. Source data were derived from the annual CIN1 cohorts.

**Table 3:** Overall 5-year risk after CIN1 diagnosed in a single calendar year, before and after restructuring to unique women.

Outcome	Before Restructuring, *n*/*N* (%)	After Restructuring, *n*/*N* (%)
CIN2+	1010/6780 (14.9)	815/5286 (15.4)
CIN3+	275/6780 (4.1)	227/5286 (4.3)
Cancer	14/6780 (0.2)	10/5286 (0.2)

Abbreviations: CIN1, cervical intraepithelial neoplasia grade 1; CIN2+, cervical intraepithelial neoplasia grade 2 or worse; CIN3+, cervical intraepithelial neoplasia grade 3 or worse. “Before restructuring” includes all annual CIN1 observations, allowing the same woman to contribute more than once across index years. “After restructuring” includes unique women only, with each woman counted once. Percentages are calculated using the number of observations or women in each dataset as the denominator.

**Table 4:** Five-year risk after CIN1 diagnosed in two consecutive years, before and after restructuring to unique women.

Analysis	*N*	CIN2+, *n* (%)	CIN3+, *n* (%)	Cancer, *n* (%)
Before restructuring (all overlapping two-year windows)	836	113 (13.5)	26 (3.1)	1 (0.1)
After restructuring (unique women in the full period)	697	100 (14.3)	24 (3.4)	1 (0.1)

Abbreviations: CIN1, cervical intraepithelial neoplasia grade 1; CIN2+, cervical intraepithelial neoplasia grade 2 or worse; CIN3+, cervical intraepithelial neoplasia grade 3 or worse. “Before restructuring” includes all overlapping two-year CIN1 windows, allowing the same woman to contribute more than once. “After restructuring” includes unique women only, with each woman counted once across the full study period. Follow-up for CIN2+, CIN3+, and cancer started after the second consecutive year with CIN1. Percentages are calculated using the number of observations or women in each analysis as the denominator.

**Table 5:** Comparison of person-based 5-year risk after CIN1 diagnosed in a single calendar year versus two consecutive calendar years.

Exposure Group	Unique Women (*N*)	CIN2+, *n* (%)	CIN3+, *n* (%)	Cancer, *n* (%)
CIN1 diagnosed in a single calendar year	5286	815 (15.4)	227 (4.3)	10 (0.2)
CIN1 diagnosed in two consecutive calendar years	697	100 (14.3)	24 (3.4)	1 (0.1)

Abbreviations: CIN1, cervical intraepithelial neoplasia grade 1; CIN2+, cervical intraepithelial neoplasia grade 2 or worse; CIN3+, cervical intraepithelial neoplasia grade 3 or worse. Percentages are calculated using the number of unique women in each exposure group as the denominator. The group with CIN1 diagnosed in a single calendar year includes women with histologically confirmed CIN1 diagnosed in a single calendar year, whereas the group with CIN1 diagnosed in two consecutive calendar years includes women with histologically confirmed CIN1 diagnosed in two consecutive calendar years. These person-based estimates show that CIN1 diagnosed in two consecutive calendar years was not associated with a higher subsequent 5-year risk than CIN1 diagnosed in a single calendar year.

## Data Availability

The data presented in this study are available on request from the corresponding author. The dataset contains individual-level health information from the SymPathy laboratory information system (University Hospital of North Norway) and cannot be made publicly available due to privacy and legal restrictions. Aggregated data are included in the article. De-identified data underlying the results may be available from the corresponding author upon reasonable request, subject to applicable approvals and data access permissions.

## Abbreviations

The following abbreviations are used in this manuscript:

ACC	Adenocarcinoma of the cervix
ACIS	Adenocarcinoma in situ
AGC	Atypical glandular cells
ASC-H	Atypical squamous cells, cannot exclude high-grade squamous intraepithelial lesion
ASC-US	Atypical squamous cells of undetermined significance
ASC-US+	Atypical squamous cells of undetermined significance or worse
CIN1	Cervical intraepithelial neoplasia grade 1
CIN2+	Cervical intraepithelial neoplasia grade 2 or worse
CIN3+	Cervical intraepithelial neoplasia grade 3 or worse
HPV	Human papillomavirus
HSIL	High-grade squamous intraepithelial lesion
LSIL	Low-grade squamous intraepithelial lesion
SCC	Squamous cell carcinoma
SPSS	Statistical Package for the Social Sciences
UNN	University Hospital of North Norway

## References

[1] Bray F., Laversanne M., Sung H., Ferlay J., Siegel R.L., Soerjomataram I., Jemal A. (2024). Global cancer statistics 2022: GLOBOCAN estimates of incidence and mortality worldwide for 36 cancers in 185 countries. CA Cancer J. Clin..

[2] Hansen B.T., Campbell S., Nygård M. (2021). Regional differences in cervical cancer incidence and associated risk behaviors among Norwegian women: A population-based study. BMC Cancer.

[3] Lowy D.R., Solomon D., Hildesheim A., Schiller J.T., Schiffman M. (2008). Human papillomavirus infection and the primary and secondary prevention of cervical cancer. Cancer.

[4] Castellsagué X. (2008). Natural history and epidemiology of HPV infection and cervical cancer. Gynecol. Oncol..

[5] Viveros-Carreño D., Fernandes A., Pareja R. (2023). Updates on cervical cancer prevention. Int. J. Gynecol. Cancer.

[6] de Sousa Gomes M.L., Moura N.D.S., Magalhães L.D.C., Silva R.R.D., Silva B.G.S., Rodrigues I.R., Sales L.B.F., Oriá M.O.B. (2023). Systematic literature review of primary and secondary cervical cancer prevention programs in South America. Rev. Panam. Salud Publica.

[7] Wentzensen N., Chirenje Z.M., Prendiville W. (2021). Treatment approaches for women with positive cervical screening results in low- and middle-income countries. Prev. Med..

[8] Bjørge T., Engesæter B., Skare G.B., Tropé A. (2022). CervicalScreen Norway—A screening programme in transition. Nor. J. Epidemiol..

[9] Nygård M., Røysland K., Campbell S., Dillner J. (2014). Comparative effectiveness study on human papillomavirus detection methods used in the cervical cancer screening programme. BMJ Open.

[10] Haldorsen T., Skare G.B., Ursin G., Bjørge T. (2015). Results of delayed triage by HPV testing and cytology in the Norwegian Cervical Cancer Screening Programme. Acta Oncol..

[11] Nygård M., Engesæter B., Castle P.E., Berland J.M., Eide M.L., Iversen O.E., Jonassen C.M., Christiansen I.K., Vintermyr O.K., Tropé A. (2022). Randomized Implementation of a Primary Human Papillomavirus Testing-Based Cervical Cancer Screening Protocol for Women 34 to 69 Years in Norway. Cancer Epidemiol. Biomark. Prev..

[12] Bjørge T., Støer N.C., Hverven S.K., Nygård M., Tropé A., Engesæter B. (2025). Risk of high-grade cervical lesions in the second round of primary human papillomavirus testing in CervicalScreen Norway: A population-based cohort study. Int. J. Cancer.

[13] Leinonen M., Nieminen P., Kotaniemi-Talonen L., Malila N., Tarkkanen J., Laurila P., Anttila A. (2009). Age-specific evaluation of primary human papillomavirus screening vs conventional cytology in a randomized setting. J. Natl. Cancer Inst..

[14] Castle P.E., Gage J.C., Wheeler C.M., Schiffman M. (2011). The clinical meaning of a cervical intraepithelial neoplasia grade 1 biopsy. Obstet. Gynecol..

[15] Kalof A.N., Cooper K. (2007). Our approach to squamous intraepithelial lesions of the uterine cervix. J. Clin. Pathol..

[16] Baasland I., Bjørge T., Engesæter B., Tropé A., Opdahl S. (2025). Cervical intraepithelial neoplasia grade 1 and long-term risk of progression and treatment. PLoS ONE.

[17] Kyrgiou M., Athanasiou A., Paraskevaidi M., Mitra A., Kalliala I., Martin-Hirsch P., Arbyn M., Bennett P., Paraskevaidis E. (2016). Adverse obstetric outcomes after local treatment for cervical preinvasive and early invasive disease according to cone depth: Systematic review and meta-analysis. BMJ.

[18] Sørbye S.W., Arbyn M., Fismen S., Gutteberg T.J., Mortensen E.S. (2011). HPV E6/E7 mRNA testing is more specific than cytology in post-colposcopy follow-up of women with negative cervical biopsy. PLoS ONE.

[19] Bruno M.T., Pagana A., Palermo I.C., Fiore M., Siena R., Lo Giudice C., Cavallaro A.G., Mereu L. (2026). Persistent Low-Grade Squamous Intraepithelial Lesions and the Risk of Overtreatment: Evidence from Long-Term Active Surveillance. Cancers.

[20] Andreassen A.K., Mortensen E., Stenbro R., Sørensen Ø., Sørbye S.W. (2025). Digital Pathology with AI for Cervical Biopsies: Diagnostic Accuracy at the CIN2+ Threshold. Cancers.

[21] Deen S., Vreynhoef S., Gandhi N., Juliana A., Nunns D., O’Neill D. (2016). The day we started HPV triage. J. Clin. Pathol..

[22] Sørbye S.W., Fismen S., Gutteberg T.J., Mortensen E.S., Skjeldestad F.E. (2014). HPV mRNA is more specific than HPV DNA in triage of women with minor cervical lesions. PLoS ONE.

[23] Sørbye S.W., Falang B.M., Antonsen M., Richardsen E. (2026). Cervical Cytology and HPV16/18/45 mRNA Co-Testing Improve Risk Stratification in Routine Clinical Practice. Cancers.

[24] Partanen V.M., Májek O., Malila N., Törnberg S., Anttila A., Nygård M., Elfström K.M. (2024). Divergent effects of switching from cytology to HPV-based screening in the Nordic countries. Eur. J. Public Health.

[25] Kjaer S.K., Nygård M., Sundström K., Dillner J., Tryggvadottir L., Munk C., Berger S., Enerly E., Hortlund M., Ágústsson Á.I. (2020). Final analysis of a 14-year long-term follow-up study of the effectiveness and immunogenicity of the quadrivalent human papillomavirus vaccine in women from four Nordic countries. eClinicalMedicine.

[26] Doorbar J., Quint W., Banks L., Bravo I.G., Stoler M., Broker T.R., Stanley M.A. (2012). The biology and life-cycle of human papillomaviruses. Vaccine.

[27] Tainio K., Athanasiou A., Tikkinen K.A.O., Aaltonen R., Cárdenas Hernández J., Glazer-Livson S., Jakobsson M., Joronen K., Kiviharju M., Louvanto K. (2018). Clinical course of untreated cervical intraepithelial neoplasia grade 2 under active surveillance: Systematic review and meta-analysis. BMJ.

[28] Castle P.E., Schiffman M., Wheeler C.M., Solomon D. (2009). Evidence for frequent regression of cervical intraepithelial neoplasia-grade 2. Obstet. Gynecol..

[29] Moscicki A.-B., Ma Y., Wibbelsman C., Darragh T.M., Powers A., Farhat S., Shiboski S. (2010). Rate of and risks for regression of cervical intraepithelial neoplasia 2 in adolescents and young women. Obstet. Gynecol..

[30] Lycke K.D., Kahlert J., Damgaard R.K., Overgaard Eriksen D., Holten Bennetsen M., Gravitt P., Petersen L.K., Hammer A. (2023). Clinical course of cervical intraepithelial neoplasia grade 2: A population-based cohort study. Am. J. Obstet. Gynecol..

[31] Bergqvist L., Virtanen A., Kalliala I., Bützow R., Jakobsson M., Heinonen A., Louvanto K., Dillner J., Nieminen P., Aro K. (2025). Predictors for regression and progression of actively surveilled cervical intraepithelial neoplasia grade 2: A prospective cohort study. Acta Obstet. Gynecol. Scand..

[32] Feiring B., Laake I., Christiansen I.K., Hansen M., Stålcrantz J., Ambur O.H. (2018). Substantial decline in prevalence of vaccine-type and nonvaccine-type human papillomavirus (HPV) in vaccinated and unvaccinated girls 5 years after implementing HPV vaccine in Norway. J. Infect. Dis..

[33] Hashim D., Engesæter B., Skare G.B., Castle P.E., Bjørge T., Tropé A., Nygård M. (2020). Real-world data on cervical cancer risk stratification by cytology and HPV genotype to inform the management of HPV-positive women in routine cervical screening. Br. J. Cancer.

[34] Carreon J.D., Sherman M.E., Guillén D., Solomon D., Herrero R., Jerónimo J., Wacholder S., Rodríguez A.C., Morales J., Hutchinson M. (2007). CIN2 is a much less reproducible and less valid diagnosis than CIN3: Results from a histological review of population-based cervical samples. Int. J. Gynecol. Pathol..

[35] Perkins R.B., Guido R.S., Castle P.E., Chelmow D., Einstein M.H., Garcia F., Huh W.K., Kim J.J., Moscicki A.-B., Nayar R. (2020). 2019 ASCCP risk-based management consensus guidelines for abnormal cervical cancer screening tests and cancer precursors. J. Low. Genit. Tract Dis..

